# Case Report: Exploration of pacemaker-specific disease management based on a generalist-specialist collaboration model

**DOI:** 10.3389/fcvm.2025.1730765

**Published:** 2026-01-12

**Authors:** Zhijun Ge, Yao Yao, Peilin Lu, Tingting Qiu, Zhaohui Qiu

**Affiliations:** 1 Department of General Practice, Huayang Community Health Service Center of Changning District, Shanghai, China; 2 Department of Cardiology, Tongren Hospital, Shanghai Jiao Tong University School of Medicine, Shanghai, China

**Keywords:** artificial, community health service, general practice, hospital, pacemaker, specialist

## Abstract

**Background:**

The management of patients with pacemakers in the community is challenged by limited resources and the need for advanced specialty care.

**Methods:**

A remote monitoring system for pacemakers was incorporated into a generalist-specialist care model, facilitating seamless collaboration between general practitioners and cardiologists. A case of pacing-induced cardiomyopathy managed through this model was presented in this study.

**Results:**

The patient received timely diagnosis and treatment, including an upgrade to a cardiac resynchronization therapy pacemaker device with remote monitoring capabilities, which significantly improved symptoms and quality of life.

**Conclusion:**

Supported by new technologies, the generalist-specialist collaboration model offers an effective approach for managing patients with pacemakers, enhancing continuity of care and optimizing healthcare resource utilization.

## Introduction

In December 2020, the Shanghai Municipal Health Commission issued the *Shanghai municipality's plan for promoting the construction of community hospitals* (1). This initiative aimed to strengthen the integration of generalist and specialist care and enhance the foundational role of community health service centers in primary diagnosis and treatment, timely referrals, and chronic disease management. In 2023, the Shanghai Municipal Government issued the *implementation plan for further improving the city's community health service capacity* (2), focusing on improving infrastructure at community health institutions and enhancing the diagnostic techniques and comprehensive capabilities at the grassroots level. The implementation of these policies promoted collaboration between specialists from general hospitals and general practitioners. This resulted in the establishment of generalist-specialist outpatient clinics, which provided strong policy support for advancing the diagnosis and treatment capabilities of communities. However, the operational models for such clinics remain inconsistent, lacking standardization and institutionalization (3). In addition, the generalist-specialist collaboration has not fully developed its distinctive characteristics (4). Moreover, this model has neither achieved differentiation from routine general practice nor effectively integrated into the broader community-based primary health care system.

Cardiac implantable electronic devices (CIEDs), commonly referred to as pacemakers, have demonstrated a consistent rise in implantation rates, leading to a growing burden of routine follow-up care (5). Evidence from related studies indicates that follow-up and device programming for patients with pacemaker implantation in cardiology departments in China are often compromised by issues such as missed routine visits and delayed management of abnormal events (6). In the community setting, the accessibility of healthcare resources and high patient compliance make remote follow-up for pacemakers a viable option. In 2019, the cardiology team at Tongren Hospital Shanghai Jiao Tong University School Of Medicine successfully obtained approval from the Shanghai Municipal Health Commission for the advanced and appropriate technology promotion project, titled *Establishment and Promotion of a Community-Based Follow-Up Management System for Cardiac Implantable Electronic Devices* (7). This initiative established China's first community-based remote follow-up management service system for CIEDs, jointly implemented by community-based general practitioners and cardiologists from higher-level hospitals. Briefly, by virtue of remote follow-up devices such as the CareLink Express Mobile system, data could be collected at the community level and transmitted to cardiology departments for evaluation and analysis (8).

In this paper, we analyzed and discussed the diagnosis and treatment process of a patient with pacing-induced cardiomyopathy (PICM) who underwent long-term follow-up at the generalist-specialist outpatient clinic of Huayang Subdistrict Community Health Service Center (hereinafter referred to as the Center). This case study shared insights on how new technologies and services were introduced to facilitate the comprehensive management of new specialty diseases in the community, providing a reference for the integrated generalist-specialist healthcare model.

## Case presentation

On June 15, 2023, an 88-year-old female patient visited our center - general practice - specialist outpatient department due to persistent chest tightness and breathing difficulties that had lasted for 3 months after a pacemaker implantation surgery 6 years ago. In 2017, the patient had repeatedly experienced transient vision loss and palpitations. 24 h ambulatory electrocardiogram monitoring revealed sinus bradycardia with sinus pauses (the longest pause was 9.1 s, with a total of 110 occurrences), and subsequently she underwent a dual-chamber rate-regulating pacemaker implantation surgery, which completely relieved her symptoms. Subsequent annual follow-up results were all normal. Since March 2023, the patient gradually developed symptoms of decreased exercise tolerance, chest tightness, breathing difficulties, and bilateral lower extremity edema. She was diagnosed with heart failure at another hospital and received diuretics and standard heart failure treatment, with symptoms alleviated.

In June 2023, due to deterioration of respiratory infection, the patient experienced significant coughing, expectoration, and severe breathing difficulties. The patient had a six-year history of leukemia, with well-controlled condition, and no history of hypertension, diabetes, hyperlipidemia, smoking, or alcohol consumption. Physical examination showed drowsiness, bilateral lower lung moist rales, a leftward extension of the dull heart area, a heart rate of 60 beats per minute, regular rhythm, no murmur, and pitting edema of both lower extremities. Laboratory tests showed an increase in white blood cells to 14.48 × 10^9^/L, an increase in C-reactive protein to 188 mg/L, a significant increase in N-terminal pro-B-type natriuretic peptide (NT-proBNP) level (2,099 pg/mL), while cardiac troponin I remained within the normal range. Chest x-ray showed pulmonary infection and cardiac enlargement. Electrocardiogram (ECG) showed bilateral atrial enlargement, and echocardiography showed mild to moderate aortic valve regurgitation, with a left ventricular ejection fraction of 59.8%. The patient was diagnosed with pulmonary infection, heart failure (New York Heart Association classification II - III), and the postoperative state after dual-chamber pacemaker implantation. Although antibiotics and diuretics improved the respiratory symptoms, chest tightness and edema persisted. Importantly, equipment examination showed a right ventricular pacing load of 99.8%, accompanied by significant QRS wave broadening, strongly suggesting PICM. After multidisciplinary discussion and obtaining the patient's informed consent, the patient was referred to the installation of a cardiac resynchronization therapy pacemaker (CRT-P, Viva CRT-P C5TR01, Medtronic), equipped with left bundle branch pacing (LBBP) and remote monitoring functions.

The diagnosis of PICM in this patient was based on the following points: (1) Long-term extremely high right ventricular pacing load: The programmed data showed that the proportion of right ventricular pacing was as high as 99.8%, far exceeding the common threshold for PICM (>40%–50%); (2) New-onset clinical symptoms and signs of heart failure: including exertional dyspnea, decreased exercise tolerance, and bilateral lower extremity edema; (3) Elevated biomarkers: NT-proBNP significantly increased (2,099 pg/mL), suggesting increased ventricular wall stress; (4) Electrocardiogram and echocardiogram evidence: The electrocardiogram showed a significantly widened QRS wave (180 ms); Postoperative echocardiography (not shown) suggested left ventricular enlargement, with a left ventricular ejection fraction (LVEF) of 59.8%, which was significantly lower than its previous stable state. During the diagnosis process, we excluded other common causes that could lead to heart failure. Since the patient had no history of coronary artery disease, no ischemic symptoms, normal myocardial troponin levels, and no ischemic changes were found on the electrocardiogram, ischemic heart disease was ruled out. Based on the patient's medical history and previous imaging examination results, hypertension-induced heart disease, diabetes-induced heart disease, and primary dilated cardiomyopathy were excluded. Although there was mild to moderate aortic valve regurgitation, its severity was insufficient to explain the rapid deterioration of the condition. The patient's initial pulmonary infection symptoms improved after anti-infection treatment, but heart failure persisted, further supporting PICM as the main cause.

Collectively, these findings supported PICM as the most plausible and unifying diagnosis given the long-term 99.8% RV pacing burden, new heart failure symptoms, biomarker elevation, and echocardiographic evidence of ventricular remodeling.

**Figure 1:**
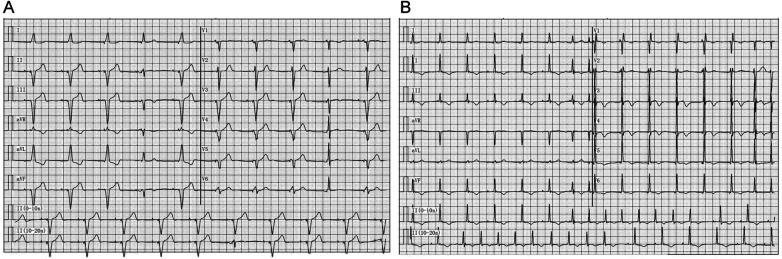
Electrocardiographic findings before and after device upgrade. **(A)** Electrocardiogram recorded on July 25, 2023, prior to the upgrade procedure. **(B)** Electrocardiogram recorded on July 27, 2023, after successful upgrade to a cardiac resynchronization therapy pacemaker (CRT-P) with left bundle branch pacing (LBBP).

Electrocardiogram examinations were conducted before and after the equipment upgrade (Figure 1). The electrocardiogram recorded on July 25, 2023 (Figure 1A) showed that before the upgrade of the CRT-P device, the heart rate of the atrium and ventricle was 60 beats per minute, the P-R interval was 178 ms, the QRS wave width was 132 ms, the QT interval and QTc interval were 446 ms and 440 ms respectively, which was in line with the rhythm of ventricular pacing.

The electrocardiogram recorded on July 27, 2023 (Figure 1B) showed that after successfully upgrading to a cardiac synchronous therapy pacemaker with left bundle branch pacing, the heart rate of the atrium and ventricle increased to 99 beats per minute, the P-R interval was prolonged to 216 ms, the QRS wave duration was shortened to 80 ms, the QT interval and QTc interval were 364 ms and 468 ms respectively, indicating that the ventricular electrical synchrony had improved after the intervention. After the pacemaker upgrade, the CRT-P device was set to DDD biventricular pacing mode, with right ventricular pacing occurring 50 ms before left ventricular pacing. The lower limit frequency was 70 beats per minute. The right atrium/right ventricle output voltage was 3.5 V/0.4 ms, the left ventricle output voltage was 4.0 V/0.4 ms, and the sensing sensitivity was 0.3 mV. No sensor functions related to heart rate response were activated after the follow-up period, and no changes were made to the pacing parameters or the minimum heart rate limit.

Within three months, the patient's chest tightness and edema subsided. Within six months, the patient's symptoms were completely relieved and their quality of life improved. This functional recovery was parallel to the rapid and continuous decline of the cardiac biomarker NT-proBNP. The duration of the QRS complex shortened from 180 milliseconds to 110 milliseconds, indicating an improvement in electrical synchrony after performing CRT-P and combining left bundle branch pacing. The OptiVol index derived from remote monitoring remained within the normal range, indicating effective management of fluid status. No significant abnormal heart rhythms or device failures were detected through remote transmission (Table 1). The patient demonstrated extremely high compliance with the prescribed drug treatment plan and the regular remote monitoring transmission. This was attributed to the continuous support of the community general practitioner. The device was well tolerated and no discomfort or side effects related to stimulation were observed. Importantly, during the entire six-month follow-up period, no adverse or unexpected events occurred, such as lead detachment, infection, or hospitalization due to heart failure.

**Table 1:** Postoperative follow-up data and changes in clinical symptoms.

Time point	NT-proBNP (pg/mL)	QRS duration (ms)	Average daily activity time (hours/day)	OptiVol index	Clinical symptoms
Before surgery (June 2023)	2,099	180	<1 h	Not applicable	Significant chest tightness, dyspnea, and bilateral lower limb edema
1 month after surgery (August, 2023)	1,450	140	2.5 h	Within normal range	Symptoms partially relieved
3 months after surgery (October 2023)	850	120	3.2 h	Within normal range	Chest tightness and edema resolved
6 months after surgery (January 2024)	450	110	3.6 h	Within normal range	Complete symptom resolution; improved quality of life

NT-proBNP, N-terminal pro b-type natriuretic peptide; OptiVol index, indicator for fluid accumulation used in device monitoring.

## Discussion

**Figure 2:**
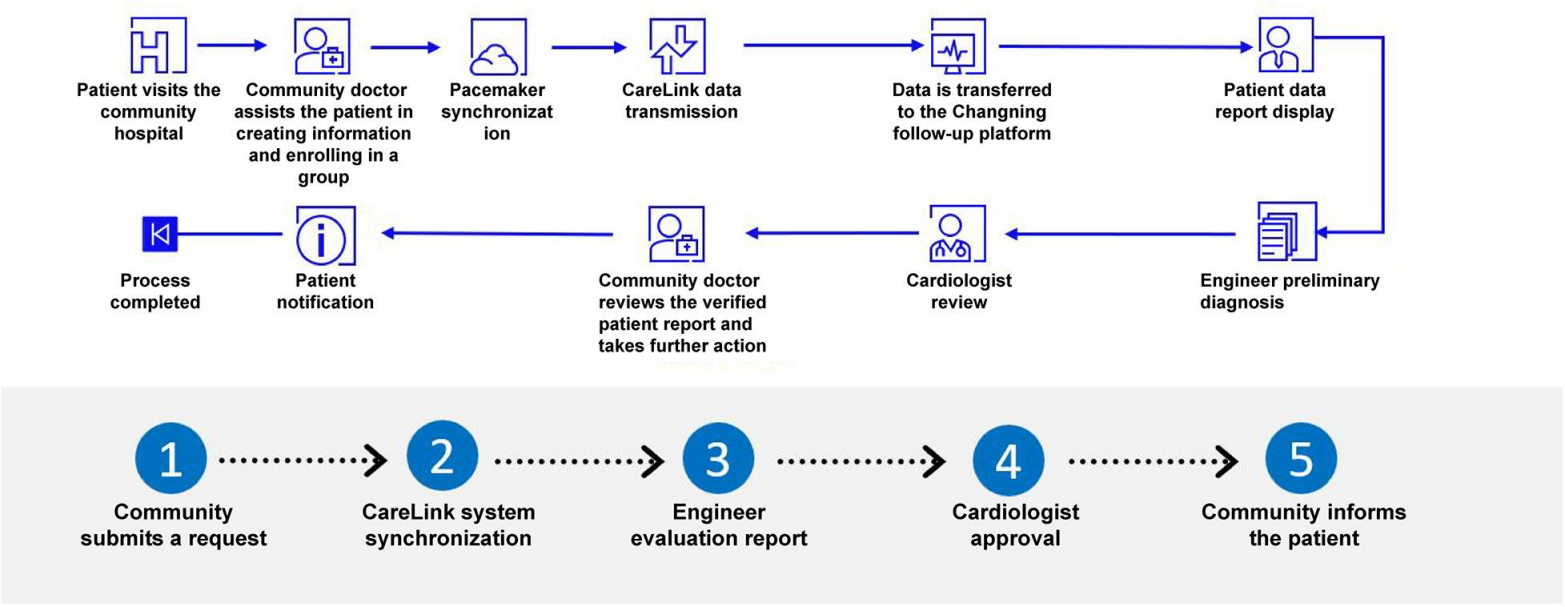
Community-based remote follow-up management service system for cardiac implantable electronic devices.

This report describes a typical case of PICM, which is significant in demonstrating how a structured collaboration model between general practitioners and specialists can enhance the timeliness, continuity, and quality of pacemaker follow-up in a real community setting. The patient had an extremely high right ventricular pacing load (99.8%), but did not undergo regular hospital evaluations, highlighting a common deficiency in the traditional follow-up system. In the collaborative model, the general practitioner (GP) detected worsening symptoms, completed a preliminary assessment, and promptly coordinated a referral to the cardiology specialist team, thereby reducing the diagnostic delay and enabling timely upgrading of the CRT-P device to adopt left bundle branch pacing. This contrasts sharply with the traditional hospital-centered workflow, where follow-up is infrequent, data continuity is limited, and patient non-compliance often leads to delayed diagnosis of PICM. After the referral, the patient benefited from a simplified process, where the pre-referral examinations were completed, which facilitated efficient surgical preparation. Postoperatively, remote monitoring provided continuous monitoring of the device and physiological status (Figure 2), enabling the GP to detect changes in NT-proBNP, QRS duration, and activity levels, and coordinate drug optimization with the cardiologist. The treatment process of this case not only demonstrated the advantages of the collaborative model, but also revealed the crucial role of precise pacing program control in it. The preoperative electrocardiogram of the patient showed a wide QRS wave pattern at the basic pacing frequency of 60 bpm, indicating significant electrical-mechanical asynchrony. This was both the pathophysiological basis of PICM and might have been exacerbated by insufficient heart rate support. Postoperatively, the intervention we implemented was twofold: on the one hand, by upgrading to CRT-P and adopting left bundle branch area pacing, we fundamentally corrected the asynchrony and significantly narrowed the QRS wave; on the other hand, we increased the lower limit frequency of the pacemaker from 60 bpm to 70 bpm, aiming to provide more adequate base heart rate support for it. The observed heart rate of 99 bpm on the postoperative ECG, which exceeded the programmed lower rate, reflects the device's normal atrial tracking function in DDD mode rather than sensor-driven acceleration, confirming appropriate device operation. Therefore, the rapid improvement of the patient's symptoms was the result of the combined effect of “physiological re-synchronization” and “adaptive frequency support”. This suggests that in the comprehensive collaborative management, the collaboration between community general practitioners and cardiac specialists should go beyond simple referral and extend to the joint formulation and follow-up of individualized treatment strategies including the optimization of pacing parameters.

**Figure 3:**
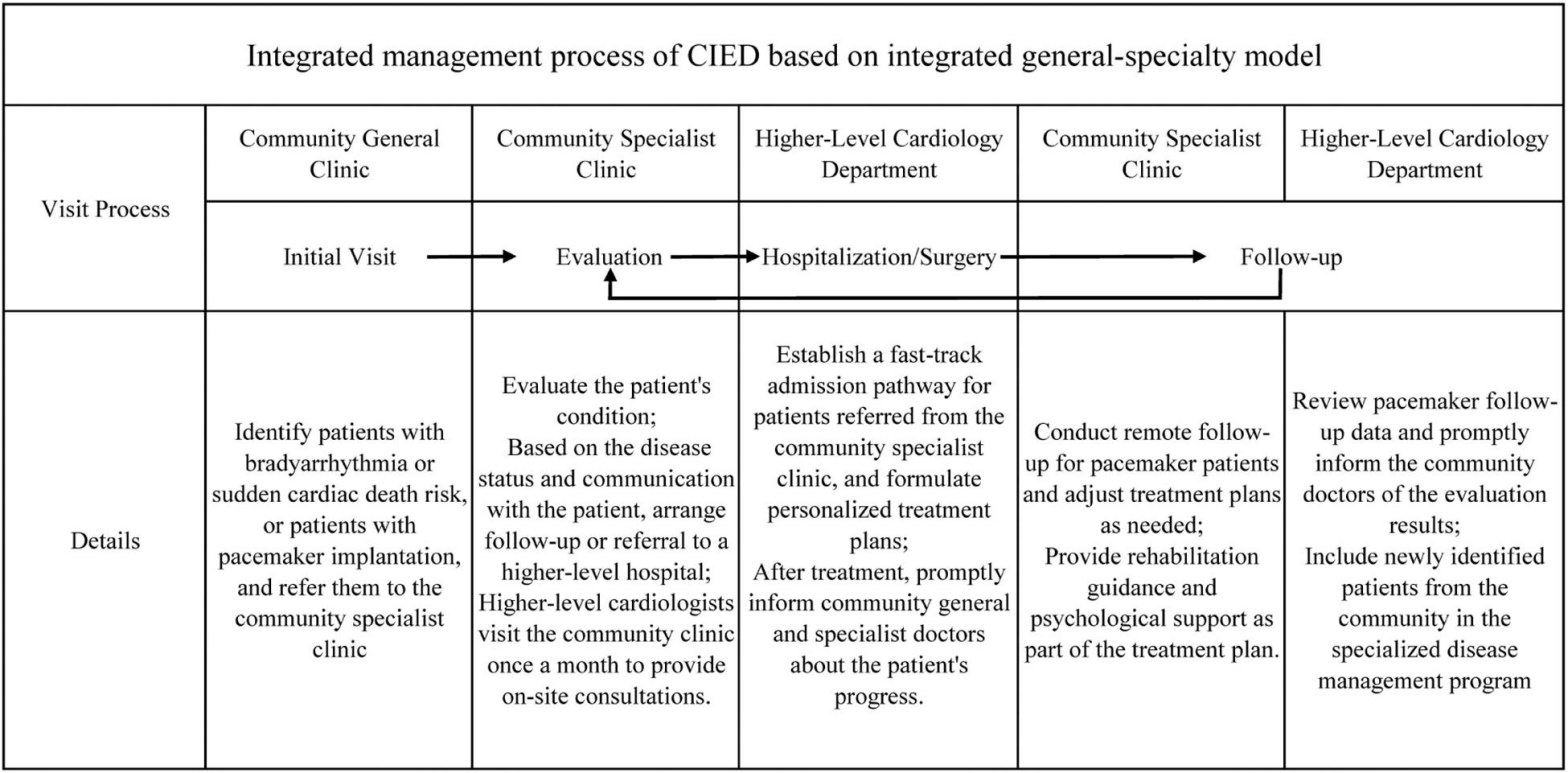
Integrated management process of cardiac implantable electronic devices based on the generalist-specialist collaboration model.

The cooperation model between general practitioners and specialists has been implemented globally, and our country also encourages the development of new types of general-practice-specialist collaboration models (9, 10). During the treatment of this patient, we established a community-based management process for pacemaker-related diseases (Figure 3), demonstrating how coordinated management can transform pacemaker care from periodic assessment to an integrated and longitudinal approach.

In addition to the clinical benefits observed in this case, we must also be aware of the potential risks associated with the upgrade surgery. Compared to the initial pacemaker implantation procedure, the complication rate of the upgrade surgery (especially the CRT-P/CRT-D surgery involving the implantation of a new left ventricular electrode) is higher. Common perioperative and postoperative complications include lead displacement (11), phrenic nerve stimulation (12), and potential device infection leading to lead extraction (13). When promoting this collaborative model and recommending that patients upgrade to a physiological pacemaker, general practitioners and cardiologists must jointly inform patients and their families of the benefits and potential risks of the surgery. During community remote monitoring and follow-up, general practitioners should also be alert for new symptoms in patients that may indicate these complications (such as hiccups, local pain, rash, fever, etc.), so as to promptly initiate a specialist consultation.

Apart from cardiac resynchronization therapy (CRT), conduction system pacing (CSP) has also become a physiological alternative for the management and prevention of PICM. Conduction system pacing (CSP), including His bundle pacing (HBP) and LBBP, achieves true physiological synchronization by directly stimulating the inherent conduction system of the heart. The latest 2025 ESC/EHRA consensus statement officially recognizes CSP as a reasonable alternative or preferred option for CRT in certain situations, including the treatment and prevention of PICM (14, 15). In this case, the introduction of LBBP during the upgrade process may help restore ventricular synchrony and facilitate the patient's subsequent functional recovery. Further research is needed to determine how CSP and CRT can be best integrated into community care pathways.

Although the integrated model demonstrates clear benefits, its implementation must be understood in the context of existing challenges within the conventional system. A major limitation of the traditional “bidirectional referral” pathway is the lack of timely and structured communication between community clinics and hospitals, which often results in fragmented care and delays in clinical decision-making (16–18).

This collaborative model directly overcomes these obstacles by replacing the temporary individual connections with structured team channels. This model ensures that referral information is pre-coordinated and enables two-way information flow between the patients. This directly eliminates the communication barriers that traditionally hindered the operation of the two-way referral mechanism, thereby enhancing efficiency and trust. Remote monitoring further strengthens this collaborative model and has been successfully applied in multiple international medical fields (19, 20). It is worth noting that although the remote monitoring system can autonomously detect abnormal conditions such as excessive pacing load in the right ventricle, its effectiveness depends on the activation mechanism and patient compliance. In this case, the remote monitoring function was not activated when the patient visited the clinic. The collaborative model does not replace remote monitoring but rather operationalizes it - by embedding this technology at the professional community level, ensuring that alerts can be promptly and appropriately handled. This can reduce the burden on hospital systems, decrease the number of patient visits, and ensure adequate follow-up. Therefore, remote monitoring has evolved from an independent tool to a proactive safety mechanism component integrated into community medical services.

This study and the proposed management model have several limitations that need to be candidly acknowledged. Firstly, this study takes a well-managed successful case as the core example. Although this research design can vividly illustrate the potential operational process and value of the model, it inevitably has selection bias and fails to reflect all the situations that may be encountered in real-world practice (such as non-compliance with remote monitoring, loss to follow-up after referral, or patients with extremely complex comorbidities). Therefore, the research findings are mainly exploratory and suggestive, and their universality and efficacy need to be verified in the future through prospective, multicenter, and large-sample cohort studies. Secondly, the smooth implementation of this model highly depends on specific technical conditions (such as compatible remote monitoring devices) and specific organizational collaboration foundations (such as close hospital-community partnerships). Data barriers between different brand devices, varying levels of informatization in community institutions, and differences in medical resource distribution among regions all pose substantial challenges for the direct promotion of this model in a wider area, especially in regions with relatively scarce resources. Finally, this study mainly focuses on the optimization of management processes and the improvement of short-term clinical indicators. The impact of this collaborative model on important long-term hard endpoints (such as all-cause mortality, re-hospitalization rate for heart failure) for patients, as well as its overall cost-effectiveness analysis, still need to be evaluated through longer-term follow-up and more in-depth health economics research. These limitations clarify the boundaries of the current study and point out the direction for future research.

## Conclusion

In conclusion, this case study demonstrates that it is feasible to establish a disease management process for pacemaker-related disorders within a comprehensive doctor-specialist collaboration model that incorporates remote monitoring technology. It provides a practical example and potential framework for the management of elderly pacemaker implantation and patients with complex specialized diseases in resource-limited community settings. Further research with larger sample sizes and controlled designs is needed to verify the effectiveness, generalizability, and long-term impact of this collaborative approach.

## Data Availability

The original contributions presented in the study are included in the article/Supplementary Material, further inquiries can be directed to the corresponding author.
